# Goal language is associated with attrition and weight loss on a digital program: Observational study

**DOI:** 10.1371/journal.pdig.0000050

**Published:** 2022-06-16

**Authors:** Annabell Suh Ho, Heather Behr, E. Siobhan Mitchell, Qiuchen Yang, Jihye Lee, Christine N. May, Andreas Michaelides

**Affiliations:** 1 Academic Research, Noom, Inc., New York, New York, United States of America; 2 Department of Integrative Health, Saybrook University, Pasadena, California, United States of America; 3 Department of Communication, Stanford University, Stanford, California, United States of America; University of Bayreuth: Universitat Bayreuth, GERMANY

## Abstract

Behavioral weight loss reduces risk of weight-related health complications. Outcomes of behavioral weight loss programs include attrition and weight loss. There is reason to believe that individuals’ written language on a weight management program may be associated with outcomes. Exploring associations between written language and these outcomes could potentially inform future efforts towards real-time automated identification of moments or individuals at high risk of suboptimal outcomes. Thus, in the first study of its kind, we explored whether individuals’ written language in actual use of a program (i.e., outside of a controlled trial) is associated with attrition and weight loss. We examined two types of language: goal setting (i.e., language used in setting a goal at the start of the program) and goal striving (i.e., language used in conversations with a coach about the process of striving for goals) and whether they are associated with attrition and weight loss on a mobile weight management program. We used the most established automated text analysis program, Linguistic Inquiry Word Count (LIWC), to retrospectively analyze transcripts extracted from the program database. The strongest effects emerged for goal striving language. In striving for goals, psychologically distanced language was associated with more weight loss and less attrition, while psychologically immediate language was associated with less weight loss and higher attrition. Our results highlight the potential importance of distanced and immediate language in understanding outcomes like attrition and weight loss. These results, generated from real-world language, attrition, and weight loss (i.e., from individuals’ natural usage of the program), have important implications for how future work can better understand outcomes, especially in real-world settings.

## Author summary

In efforts to improve outcomes of weight management programs such as weight loss and dropout, research has started to explore automated interventions that act based on models of digitally collected data. To build good models, it may be helpful to identify variables that are relevant for the outcomes and therefore should be considered. In this study, we explored whether the language that individuals write during a weight management program is relevant for outcomes; this question has not been studied yet. Previous research has shown that psychologically distanced language (e.g., what the situation means) is associated with better outcomes like academic performance or emotional control, while more immediate language (e.g., the details of the situation) is related to worse outcomes. We explored whether language during actual use of a program (Noom Weight) was associated with weight loss and dropout. We examined language data gathered from the program database using automatic language processing. We found that in interactions with a coach, distanced language was associated with better outcomes (less dropout and more weight loss) and immediate language was associated with worse outcomes (more dropout and less weight loss). These results raise many directions for future intervention development and highlight that this language should be considered and evaluated in the future.

## Introduction

According to recent estimates, obesity affects more than 37% of adults in the US alone and is associated with increased risk of cardiovascular and metabolic conditions [1,2]. Moderate weight loss may significantly reduce the risk of weight-related health complications [3,4]. Behavioral weight loss programs help individuals to achieve moderate weight loss through behavior change education and support [5]. Measurable outcomes of these programs include weight loss and attrition, or the extent to which individuals drop off the program before receiving the full intervention. Understanding what behavioral factors are associated with weight loss and attrition can be an initial step towards “offering special assistance, structure, therapist contact and/or a more targeted intervention for those at highest risk of dropout” in future programs, with the ultimate goal of improving outcomes [6–9]. As an example, recent research has sought to understand how to improve outcomes by leveraging continuously collected data on digital (e.g., smartphone) platforms to identify real-time moments to intervene [10–12]. Algorithmic models are built to predict real-time at-risk moments, and they are initially informed by knowledge about which characteristics or behaviors are associated with the outcomes of interest [10,13]. Data such as self-reports of eating instances or smartphone metadata (e.g., accelerometry and geospatial location) have been used in this context so far [14,13]. In this study, we explore whether a new source of data, individuals’ language, reveals associations with attrition and weight loss. Exploring these associations is a first step towards future efforts in real-time identification or intervention that, ultimately, could help to improve outcomes [10].

Specifically, in this study, we focus on individuals’ language during regular use of a weight management program. In many programs, individuals write an initial goal (e.g., “Lose 10 pounds”, “become healthier”) or interact with a coach about their goals. This investigation is theoretically and empirically motivated in two ways. First, the study is motivated by previous research on individuals’ language. According to psycholinguistic theories, individuals’ words reveal key information about their perspective, thoughts, or focus [15,16]. Decades of research have shown that individuals’ words explain significant variation in health outcomes such as the likelihood of alcohol relapse, physical and psychological health, and to some extent, weight loss [17–20]. For example, there is a positive association between the number of words individuals use related to processing and coming to new insights (e.g., “realize”, “because”) and health improvements [16]. For goals specifically, an important predictive factor may be *distanced* (vs. immediate) language. Various terms are used in the literature, such as narrative vs. categorical or analytical [21,22], or psychologically close vs. distanced [23,24]. Generally, individuals’ words range on a spectrum from immediate (e.g., focused on the details of the present moment) to distanced (i.e., focused on abstract or analytical connections between concepts) [21–25]. Work has demonstrated that immediate writing is marked by using more words tied to the present situation such as personal pronouns (e.g., “I”, “you”), present-tense words, auxiliary verbs (“is”, “are”), and discrepancy words (“should”, “would”). Distanced language, on the other hand, has more markers of abstract or analytical thinking such as articles (“the”, “a”) and prepositions (“with”, “among”) [21,23,26]. As an illustration, consider a sentence such as, “I should try harder to stick to my plan today” (immediate) compared to, “the idea of ignoring the food is stressful” (distanced). Language that has markers of distanced language (vs. immediate language) is associated with better (vs. worse) self-regulation and self-reported health in correlational and experimental work [23–26]. This is likely because focusing on the details of the present (e.g., how tasty this food smells) can deter individuals from their goals, while having a more distanced, abstract, and higher level perspective could focus one’s attention on broader motivations and reasons (e.g., why this food isn’t healthy) [27]. Therefore, the types of words individuals use, such as markers of psychological distance, could be associated with attrition and weight loss.

Second, the study is motivated by research suggesting that individuals’ written language about *goals* may provide insight into outcomes such as weight loss success and attrition. To our knowledge, there are no previous studies specifically exploring the relationship between writing about goals and attrition or weight loss. However, previous work has shown that in weight management interventions more broadly, individuals’ goals for weight loss are associated with attrition and weight loss [8,28,29], suggesting that exploring goals may be informative in the context of this study. Moreover, studies have shown that the way individuals write about their goals is associated with outcomes in other domains such as subjective well-being and academic performance [30,31]. This could be because, according to goal construal theory, the way one frames a goal influences how well one can self-regulate towards that goal; for example, as described previously, framing goals in terms of the “how” instead of the “why” may focus attention on immediate temptations rather than a higher level motivation [27,32,33]. Further, goal-setting theory stipulates that the way individuals conceive of their goals, such as how important or difficult they perceive the goal to be, influences self-regulatory behaviors and outcomes [34]. For instance, individuals show more goal persistence and success when they perceive the goal to be sufficiently difficult [34]. Based on these studies, we speculate that the way individuals conceive of, and ultimately express, their goals in writing could relate to attrition and weight loss.

Therefore, in this study, we explore whether the words individuals use to describe their goals are associated with attrition and weight loss. Additionally, the study expands on previous work by distinguishing between goal setting and goal striving, two distinct components of goals [30,35]. Goal setting describes setting an initial goal, while goal striving is the process through which goals are pursued. In this retrospective study, we leverage a unique dataset allowing us to examine the words that individuals used in goal setting and goal striving during actual use of a digital program [36]. We collected the language used in goal setting (i.e., setting their overall goal at the start of the program) and goal striving (i.e., in goal striving conversations with coaches) by users of Noom Weight, a mobile behavior change program. Using automated text analysis, we explored if this language was associated with attrition and weight loss. Since this is the first study of its kind in the domain of weight management, we used the closest equivalent studies to choose language variables and form hypotheses [19,23,37]. We hypothesized that articles, auxiliary verbs, and prepositions (i.e, markers of distanced language) would be associated with lower attrition and greater weight loss. We also hypothesized that personal pronouns, present-tense words, auxiliary verbs, and discrepancy words (i.e., markers of immediate language) would be associated with greater attrition and less weight loss. Additionally, based on the patterns found in prior studies of language on weight loss blog posts [19,37], we hypothesized that positive emotion words (“happy”), negative emotion words (“sad”), cognitive processing words (“realize”, “know”), ingestion words (“eat”, “food”), social words (“spouse”), and certainty words (“certain”, “definitely”) would be associated with lower attrition and greater weight loss. We also hypothesized that tentative words would show the opposite pattern (“maybe”, “perhaps”). We also explored future tense words since an orientation towards the future could be beneficial for goal striving [38]. We did not a priori expect different results for goal setting and goal striving.

## Methods

This was a retrospective study in which text, weight, and activity data were extracted from the Noom database and analyzed from baseline to however long participants remained on the program, up to 16 weeks, which is the length of the core program.

### Program

Noom Weight is a commercial digital behavior change program that has demonstrated aiding in clinically meaningful weight loss in both observational and randomized controlled trials [39,40]. Past studies have demonstrated moderate effect sizes for weight change on Noom that range from 0.6 to 0.99 (Cohen’s d) [41,42]. Noom’s theoretical underpinnings are cognitive behavioral therapy (CBT), motivational interviewing, and third-wave CBT techniques, which all are effective for weight loss [43–45]. When users first start the program, they are asked to write an overall goal. After the first goal, they are encouraged to write more personally meaningful goals in text fields provided after a series of “why?” questions. All the text used in this goal-setting exercise were combined (e.g., “Lose 10 pounds To be healthier To be able to enjoy possible grandchildren”). Human coaches trained in motivational interviewing and goal setting techniques help participants to break down their goal into smaller incremental goals and to talk about barriers, facilitators, and thoughts surrounding each goal. At the time of this study, coaches checked in with participants about their goal progress at least once a week in text-based messages in the mobile program. We analyzed all language participants wrote to set their overall goal and message their coach.

### Participants

**Fig 1:**
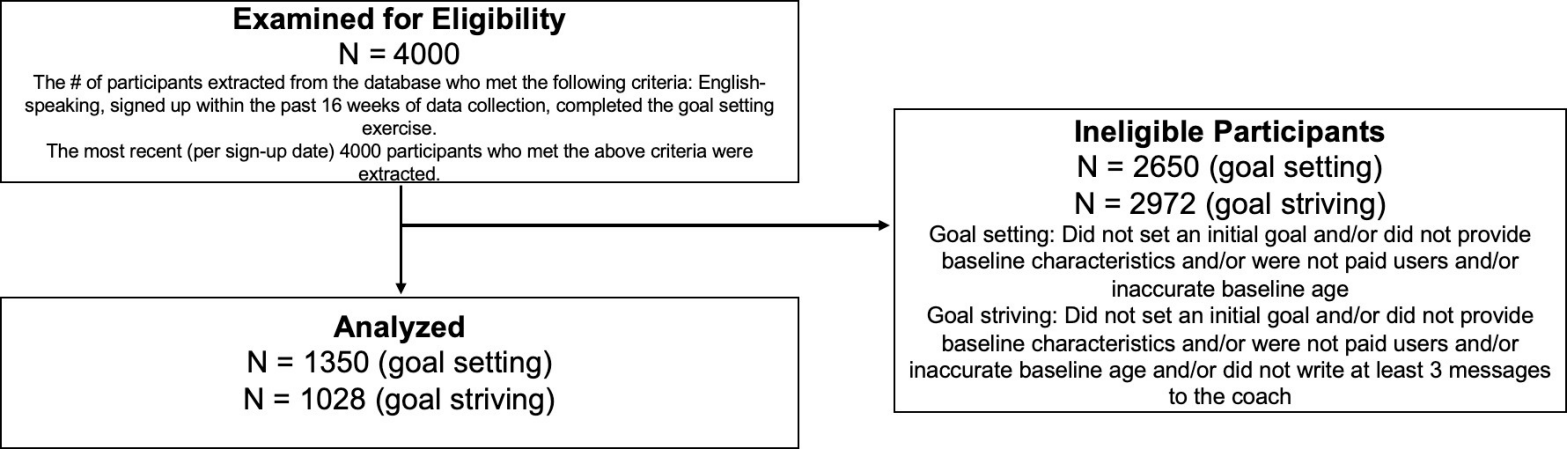
Flow diagram of inclusion and exclusion.

All participants had voluntarily signed up for the program online or in the app store due to their own desire to lose weight. The Advarra IRB approved this study. All individuals analyzed in this retrospective study provided written electronic informed consent at program sign-up for their program data to be analyzed in retrospective research; all individuals were given the option to opt out. All data, which was extracted from the program database retrospectively, were fully anonymized before analysis. Individuals were eligible for this study if they had signed up within the past 16 weeks at the time of data collection (September 2020), were English-speaking users, and had completed the goal setting exercise within the past 16 weeks. Data from the 4000 most recent signups meeting those criteria were initially extracted from the database. Out of these 4000 individuals, 1351 set an initial goal, provided all baseline characteristics of age, gender, weight, and height, and paid for the program. These criteria were chosen to ensure adequate language and baseline data for models. We focused only on paid users because it could be misleading to examine attrition in users who had no intention of paying for the program. One participant was excluded because of unrealistic responses to baseline characteristics (e.g., “0” years old). Thus, 1350 participants were included in goal setting analyses. Out of those 1350, goal striving analyses included those sent at least 3 messages to the coach (N = 1028). See Fig 1 for a diagram of inclusion.

### Measures

#### Attrition

Attrition was calculated as a dichotomous variable. Since this is a publicly available commercial program that does not have a specified end date, we used 16 weeks since this is the length of the core program. After 16 weeks, the psychoeducation shifts towards maintenance and deeper exploration of previous topics. If participants’ last week of any in-program action was equal to or greater than 16 weeks, they were coded as 0 (retained). If their last week of an in-program action was before 16 weeks, they were coded as 1 (attrited).

#### Language

Goal-setting language was defined as the language individuals used in setting their overall goal on the first day of the program. Goal-striving language was defined as the text messages sent to the coach during goal striving conversations. Transcripts from individuals’ initial goal writing exercise and all of their messages to the coach were extracted from the Noom database. The most common automated text analysis program, Linguistic Word Inquiry Count (LIWC), was used to process and analyze language. LIWC contains pre-defined categories of thousands of words that were built and validated with human coders [46]. For example, the positive emotion category contains words such as “happy” and “excited”. LIWC calculates the percentage of words in the text that fall into each category out of the total number of words used. In this way, LIWC takes into account differences in word count. The following categories were analyzed: articles (“a”), personal pronouns (“I”), prepositions (“on”), discrepancy (“should”), auxiliary verbs (“is”, “are”), present tense, future tense, positive emotion (“happy”), negative emotion (“frustrated”), ingestion (“eat”), social (“friends”), insight (“think)”, causation (“because)”, tentative (“maybe”), and certainty (“always”) words. Table 1 describes all the language categories analyzed.

**Table 1:** All language categories analyzed and the most relevant scientific concept.

Language category	Related concept	Citation
Articles	Distanced (vs. immediate) language	Pennebaker et al 2014; Nook et al 2017
Prepositions	Distanced (vs. immediate) language	Pennebaker et al 2014
Auxiliary verbs	Immediate (vs. distanced) language	Pennebaker et al 2014
Personal pronouns	Immediate (vs. distanced) language	Pennebaker et al 2014; Nook et al 2017
Present tense	Immediate (vs. distanced) language	Pennebaker et al 2014; Nook et al 2017
Discrepancy words	Immediate (vs. distanced) language	Nook et al 2017
Positive emotion	Emotion	Tausczik & Pennebaker 2010
Negative emotion	Emotion	Tausczik & Pennebaker 2010
Insight words	Cognitive processes	Tausczik & Pennebaker 2010
Causal words	Cognitive processes	Tausczik & Pennebaker 2010
Tentative words	Cognitive processes	Tausczik & Pennebaker 2010
Certainty words	Cognitive processes	Tausczik & Pennebaker 2010
Differentiating words	Cognitive processes	Tausczik & Pennebaker 2010
Ingestion words	Food/Weight	Chung et al 2010
Social words	Social relationships	Tausczik & Pennebaker 2010
Future tense	Future goal orientation	Tausczik & Pennebaker 2010

#### Weight

Participants were encouraged, but not required, to self-report their weight on the program every week. We extracted participants’ self-reported weight from the program database and calculated weight loss via the difference between baseline weight and weight at 16 weeks. For participants who did not report their weight at 16 weeks, weight values were carried forward via last observation carried forward.

### Statistical analysis

All analyses were carried out in R (version 3.6.0) with an alpha of .05. Descriptive statistics are expressed in means and standard deviations. Assumptions for linearity were checked and met. First, to assess associations with attrition and weight loss, univariate models with each language marker were conducted. All univariate models controlled for age because retained and attrited participants significantly differed on age. In addition, univariate models for weight loss controlled for baseline BMI. Logistic regressions were used to predict the likelihood of attriting, and linear regressions were used to predict the amount of weight loss. Next, because numerous language markers were analyzed, we controlled for multiple comparisons with the Benjamini-Hochberg correction with false discovery rate [47]. Finally, to assess which language categories were most associated with attrition and weight loss above others, multivariate models were conducted including covariates (age for attrition and age and BMI for weight loss) and all language markers that were significant from the univariate models after multiple comparison correction.

## Results

### Baseline characteristics

Descriptive characteristics for all participants, split by those who attrited or retained, are displayed in Table 2. Attrited and retained users did not differ on baseline BMI or gender but differed on age (range: 18 to 85). Attrited participants were younger (M = 43, SD = 13) than retained participants (M = 46, SD = 13) for both goal striving and goal setting samples. Retained users lost 5.2–5.3 kg (SD = 4.8) at 16 weeks, or 5.4–5.6% body weight (SD = 5.0%). Attrited participants, on the other hand, lost 1.9–2.0 kg (SD = 2.5) when their last weight observation was carried forward, which constituted 2.1–2.2% (SD = 2.7%).

**Table 2:** Baseline characteristics for attrited and retained participants.

	Participants included in goal striving analyses			Participants included in goal setting analyses		
	Attrited (n = 726)	Retained (n = 624)	*p*	Attrited (n = 496)	Retained (n = 525)	*p*
Program duration (weeks), mean(SD)	7.3 (3.9)	16 (0)	< .001	7.8 (3.9)	16 (0)	< .001
Age(years), mean(SD)	42.8 (12.9)	46.4 (13.1)	< .001	42.8 (13.3)	46.2 (13.2)	< .001
Baseline BMI, mean(SD)	32.4 (11.0)	33.3 (7.6)	.09	32.5 (9.8)	33.5 (7.7)	.10
Gender, n(%)			.47			.10
Female	602 (82.9%)	507 (81.3%)		425 (85.7%)	435 (87.7%)	
Male	124 (17.1%)	117 (18.7%)		71 (14.3%)	89 (17.9%)	
Weight loss (kg)	1.9 (2.5)	5.2 (4.8)	< .001	2.0 (2.5)	5.3 (4.8)	< .001
Weight loss (%)	2.1 (2.7)	5.4 (5.0)	< .001	2.2 (2.7)	5.6 (5.0)	< .001

Note: Weight loss was calculated by weight at week 16 (retained) or carrying forward observations to week 16 (attrited) for participants who entered baseline weight and at least one other weight observation.

### Goal setting language

#### Attrition

Controlling for age, participants’ tentative words during goal setting were negatively associated with attrition (odds radio [OR] = .91; 95% CI: .86, .97, p = .005). A one unit increase in tentative words was associated with a .91 times reduction in the odds of attrition. None of the other language variables significantly predicted attrition.

#### Weight loss

After adjusting for age and baseline BMI, only positive emotion words (B = -.04-, 95% CI: -.08, -0.01, p = .01) and insight words (B = -0.07, 95% CI: -.12, -0.02, p = .01) were associated with greater weight loss. However, these became not significant when adjusting for multiple comparisons.

### Goal striving language

#### Attrition

As expected, in goal striving conversations, after adjusting for age and multiple comparisons, personal pronouns, discrepancy words, present tense words, and auxiliary verbs (all markers of immediate language) were associated with higher likelihood of attrition (Table 3; all ps < .02). For example, a one-percent increase in personal pronouns was associated with a 1.13 times increase in the odds of attrition (OR = 1.13, 95% CI 1.08, 1.18; p < .001). Articles, a marker of distanced language, were associated with lower likelihood of attrition (OR = 0.86, 95% CI 0.80, 0.93; p < .001). A one-percent increase in articles was associated with a .86 times reduction in the odds of attrition. Thus, more distanced language (articles) was associated with lower likelihood of attrition, and markers of immediate language (pronouns, discrepancy words, present tense words, and auxiliary verbs) were associated with greater likelihood of attrition.

**Table 3:** Goal striving: Significant univariate age-adjusted associations between language categories and attrition during goal striving (logistic regressions).

Covariate	Related concept	B	SE	Wald	*p*	OR	95% CI
Articles	Distanced (vs. immediate) language	-0.15	0.04	-3.72	< .001**	0.86	(0.80, 0.93)
Auxiliary verbs	Immediate (vs. distanced) language	0.14	0.03	5.26	< .001**	1.15	(1.09, 1.21)
Personal pronouns	Immediate (vs. distanced) language	0.12	0.02	5.26	< .001**	1.13	(1.08, 1.18)
Present tense	Immediate (vs. distanced) language	0.09	0.02	4.15	< .001**	1.10	(1.05, 1.15)
Discrepancy words	Immediate (vs. distanced) language	0.24	0.06	4.00	< .001**	1.27	(1.13, 1.43)
Tentative words	Cognitive processes	-0.11	0.05	-2.494	.01*	0.89	(0.81, 0.97)
Future tense	Future goal orientation	-0.14	0.06	-2.26	.02*	0.87	(0.77, 0.98)

Note: For the sake of readability, results for all language categories (including non-significant results) are presented in S1 Text Table C.

* denotes significance without multiple comparison adjustment

** denotes significance with multiple comparison adjustment

A multivariate model was conducted with language categories that had been significant in univariate logistic regressions after multiple comparison adjustment (personal pronouns, discrepancy words, present tense words, articles, auxiliary verbs). In this multivariate model, personal pronouns, discrepancy words, and auxiliary verbs were significantly associated with higher likelihood of attrition (S1 Text Table D, all ps < .03). Only articles and present tense words were not associated with attrition.

#### Weight Loss

Aligning with the attrition results, for weight loss, greater use of personal pronouns, auxiliary verbs, discrepancy words, and present tense words (markers of immediate language) were associated with less weight loss in univariate regressions after adjustment for multiple comparisons, age, and baseline BMI (Table 4, all ps < .001). Articles and prepositions (markers of distanced language) as well as future tense words were associated with more weight loss (all ps < .001). In a multivariate model with all of these significant language categories, only personal pronouns, discrepancy words, and future tense words were significant (all ps < .04; S1 Text Table D). Personal pronouns and discrepancy words were associated with less weight loss, while future tense words were associated with greater weight loss.

Table 5 presents a summary of the results.

**Table 4:** Goal striving: Significant univariate age and BMI-adjusted associations between language categories and weight loss during goal striving (linear regressions).

Covariate	Related concept	B (95% CI)	SE	*t*	*p*	Cohen’s f2
Articles	Distanced (vs. immediate) language	0.26 (0.10, 0.41)	0.08	3.29	.001**	.01
Auxiliary verbs	Immediate (vs. distanced) language	-0.15 (-0.24, -0.06)	0.05	-3.21	.001**	.01
Personal pronouns	Immediate (vs. distanced) language	-0.23 (-0.31, -0.14)	0.04	-5.15	< .001**	.03
Present tense	Immediate (vs. distanced) language	-0.17 (-0.26, -0.09)	0.04	-4.04	< .001**	.01
Discrepancy words	Immediate (vs. distanced) language	-0.40 (-0.62, -0.17)	0.11	-3.47	< .001**	.01
Future tense	Future goal orientation	0.45 (0.20, 0.69)	0.12	3.61	< .001**	.01

Note: For the sake of readability, results for all language categories are presented in S1 Text Table E.

* denotes significance without multiple comparison adjustment

** denotes significance with multiple comparison adjustment. Bs represent unstandardized betas. A Cohen’s f2 of .02 is estimated to be a small effect size.

**Table 5:** Summary of the language categories showing significant associations with each type of goal language and outcome.

	Univariate Analyses		Multivariate Analyses	
	Attrition	Weight Loss	Attrition	Weight Loss
**Goal Setting**	Certainty words (-)* Tentative words (-)**	Positive emotion (+)* Insight words (+)*	–	–
**Goal Striving**	Articles (-) ** Auxiliary verbs (+) ** Personal Pronouns (+) ** Present tense (+)** Discrepancy words (+) ** Tentative words (-)* Future tense (-) *	Articles (+)** Auxiliary verbs (-)** Personal Pronouns (-)** Present tense (-)** Discrepancy words (-)** Prepositions (+)** Future tense (+)**	Auxiliary verbs (+)*** Discrepancy words (+)*** Personal pronouns (+)***	Future tense (+)*** Discrepancy words (-)*** Personal pronouns (-)***

Note: (-) denotes a negative association and (+) denotes a positive association

* denotes significance without multiple comparison adjustment

** denotes significance with multiple comparison adjustment

*** denotes significance in one multivariate analysis (with no multiple comparisons)

## Discussion

In this study, we used automated text analysis to observe if individuals’ language in goal setting and striving on a mobile program related to outcomes of attrition and weight loss. Results revealed that certain types of language are associated with attrition and weight loss. We explored the types of words (i.e., language markers) used to set short initial goals at the start of a program (goal setting) or in conversing with a coach about daily efforts to achieve those goals (goal striving). Outcome measures were attrition and weight loss. We found that the strongest effects emerged for psychologically immediate or distanced language in goal striving, with similar results for both attrition and weight loss. As hypothesized, markers of immediate language were associated with worse outcomes (i.e., higher likelihood of attrition and lower weight loss), and markers of distanced language were associated with better outcomes (i.e., lower likelihood of attrition and higher weight loss) in univariate models. In multivariate models, some markers of immediate language predicted lower likelihood of attrition and weight loss over and above markers of distanced language. Additionally, future tense words were associated with greater weight loss in both univariate and multivariate models. The results for goal setting were more limited than for goal striving. Unexpectedly, tentative language in goal setting was associated with lower likelihood of attrition. For weight loss, goals set with more positive emotion and insight words were associated with better outcomes. Overall, effects for goal striving may have been stronger than for goal setting because many more words are used in conversations with a coach as opposed to setting an initial goal. We also speculate that in consistent conversation with a coach about one’s goals and progress, there is greater opportunity to engage in more distanced, analytical thinking (e.g., overall patterns) or to focus attention on the immediate (e.g., what I should do today) than in setting one initial goal. Below, we will first describe the results for goal striving, followed by the findings for goal setting.

### Goal striving

#### Attrition and weight loss

As noted previously, in univariate analyses, we found that markers of psychologically immediate language were associated with worse outcomes and markers of distanced language were associated with better outcomes for both attrition and weight loss. Our findings closely corroborate previous work. Previous studies have consistently found that markers of immediate language are associated with worse outcomes and markers of distanced language associated with better outcomes such as emotional regulation, college GPA, and self-reported health [21,23,25]. Because we found the same pattern within the domain of weight management, these language markers (pronouns, discrepancy words, auxiliary verbs, present tense words, articles, and prepositions) may be associated with outcomes across other behavior change domains. Future work should ascertain the extent to which these language markers are informative for other behavior change domains involving different types of behaviors than those relevant to weight management. Future work should also directly compare associations for multiple outcomes and behavior change domains to ascertain when this language is informative and when it is not.

Going beyond univariate models, when significant language markers were combined together in multivariate analyses, markers of immediate language remained significant while markers of distanced language were no longer significant. This result occurred for both attrition and weight loss. Previous studies had either combined immediate and distanced language markers into one factor or examined associations between each individual marker and outcomes [21,23], which does not provide information about the relative importance of each marker compared to others. Our multivariate results suggest that psychological distance could be helpful to some extent, but focusing on the immediate is more strongly related to negative outcomes. This notion aligns with previous work which, taken altogether, suggests that focusing on the present is relevant for and can undermine weight management efforts. This body of work is based on the idea that in current Western obesogenic environments, an overabundance of highly available and palatable foods, along with reward-based neurobiological drives, create many opportunities for overwhelming immediate temptations [48,49]. Smells or pictures in the immediate vicinity can even activate neural pathways related to eating that can spur eating behavior [50]. Focusing on the immediate present, with its temptations with powerful immediacy, could impair effortful control of eating behavior, which is an important component of weight management [51,52]. Therefore, it is possible that immediate language was a stronger signal of outcomes than distanced language because of the many instances of managing, avoiding, and preventing immediate food-based temptations during weight management and the importance of controlled eating behaviors in weight-related behavioral change. However, future research should test this explanation.

The results explained above applied to both attrition and weight loss. While the results were largely similar for both outcomes, there were a few minor differences. First, in univariate analyses, prepositions were significant for weight loss but not for attrition. Prepositions are not included in all conceptualizations of distanced language [23], so it is possible that prepositions show weaker or less consistent effects than other markers that are better understood to be a marker of distanced language. Second, future tense words were associated with greater weight loss in both univariate and multivariate models, but were not as strongly associated with attrition. To our knowledge, this is the first time that future tense words have been found to be associated with greater weight loss. Research suggests that an orientation towards the future can reduce impulsivity (e.g., overeating episodes), and that future tense words are associated with fewer risky behavioral outcomes [53,54]. One example of an orientation towards the future is delayed discounting, which is the tendency to prefer short term gain over long term rewards [55]. According to a burgeoning literature, greater delayed discounting (i.e., valuing immediate rewards more than longer-term rewards) is associated with weight gain, and reduced delayed discounting is associated with weight loss [55,56]. Studies suggest that reflecting on the future via episodic future thinking (e.g., imagining future outcomes of present actions) may reduce delayed discounting [53,57]. Further, there is initial evidence that future tense words are used when reflecting on the future [58]. It is possible, then, that future tense words exert effect on weight loss *through* episodic future thinking and delayed discounting, in which future tense words are used when individuals reflect on the future, which reduces delayed discounting, which then increases weight loss. This mediational pathway should be tested in future research. However, another study showed that individuals randomly assigned to focus on the future, such as one’s future ideal outcomes, did not experience greater weight loss than those who were assigned to focus on the past (e.g., one’s past self or behaviors) [59]. Clearly, more work is needed to ascertain how future tense words are related to weight loss.

### Goal setting

Results for goal setting were not significant after adjusting for multiple comparisons; however, we describe possible explanations for the results in more detail below in case they can still inform future studies or predictive algorithmic models.

#### Attrition

To our knowledge, this is the first study to report that tentative language in forming goals was associated with lower likelihood of attrition. In the absence of similar studies, we speculate that these results could be related to intrinsic goals. We speculate that the type of writing exercise lent itself to a positive relationship between tentative language and attrition. When joining this program, as seen in previous studies of other programs [60,61], individuals usually have a clear weight goal in mind (e.g., lose 10 pounds). Thus, we would not expect tentative language to be used when setting the first layer of their goal. Instead, we would expect tentative language to appear in subsequent higher-level responses as individuals responded to a series of “why” questions (e.g., “lose 10 pounds”, “why?”, “to be healthier”) about the deeper reasons they want to achieve their goal. As a result, tentative language was most likely used when participants explored other personally relevant goals than a specific goal weight (e.g., “perhaps feeling more confident”, “maybe having more energy”, “to play with and enjoy possible grandchildren”). Tentative language tends to be used when pondering or thinking through a new idea or concept [22], and tentative language was significantly correlated with insight words (“realize”) in this data (r = 1.8, p < .001). Thus, we speculate that tentative language may have been used more in exploring higher-level, more intrinsic (i.e., deeply personally relevant) goals. Intrinsic goals are associated with lower attrition in behavior change interventions [62,63]. Future work should test this potential explanation and explore whether tentative language in other types of behavior change programs predicts attrition.

#### Weight loss

For weight loss, goal setting language of positive emotion (“happy”, “satisfied”) and insight words (“realize”, “think”, “know”) were associated with greater weight loss in univariate models. These results are consistent with work suggesting that positive emotion and insight words, in combination, may reflect broadened cognitive and behavioral flexibility [64]. This program is based on cognitive behavioral therapy (CBT) and third-wave CBT, which seek to improve cognitive flexibility, helping individuals to reframe their goals and experiences in broader and more accepting contexts [65,66]. Greater cognitive flexibility is also more generally associated with greater weight loss [67,68]. Thus, it is possible that positive emotion and insight words reflected cognitive flexibility and broadening, which was then related to weight loss. Future work should test this potential mediational pathway, such as exploring whether initial goals with more insight and positive emotion words lead to more weight loss through greater cognitive flexibility.

### Other findings

Significant results did not emerge for expected language categories like positive emotion, negative emotion, ingestion words (“eat”, “food”), and social words, and all cognitive processing words except for insight and tentative words. These variables were chosen based on the closest relevant study, which found associations between these words and attrition using language sourced from online weight loss blogs [37]. The difference in results could be because weight loss blogs are affiliative spaces in which individuals want to connect with others and express their thoughts and emotions; the more emotion is expressed, the more engagement on the blog increases [69–71]. Therefore, perhaps writing or processing emotionally is more closely connected to retaining if defined in terms of continuing to write on the blog. In contrast, retaining on a weight loss program may not be as closely tied to emotional expression and cognitive processing. In our past work, we found in a sample of individuals who were active, and thus retained, for 16 weeks, negative emotion words significantly increased over time [36]. This along with the current findings suggest that emotion is not a strong indicator of attrition in this context. Future work should explore language used on a variety of programs and settings. As weight management programs grow in popularity and reach, more research should investigate the language that can help to understand how to improve outcomes like attrition and weight loss.

### Practical implications

Now that overall associations have been found between immediate or distanced language and outcomes like weight loss and attrition, predictive models can include and further examine these variables to identify high-risk moments or individuals based on their language. Beyond identification, just-in-time interventions could also provide real-time intervention based on language. In previous studies, just-in-time interventions have provided an in-time intervention encouraging participants to change their behavior in some way [12]. In this case, the intervention could be to encourage individuals to write in a less immediate and more distanced way. In previous studies using experimental designs, individuals were randomly assigned to write in more distanced or more immediate ways despite their natural writing tendencies. Importantly, individuals in the distanced writing conditions showed evidence of better emotional regulation than individuals in the immediate conditions [23]. It may be possible, then, that asking individuals to write in more distanced (and less immediate) ways may improve weight loss and attrition. Future research should use experimental designs to evaluate this question. Further, since previous studies have only compared distanced writing to immediate writing, future research should also directly compare conditions that have been asked to write naturally to conditions asked to write with more distance or immediacy in order to ascertain whether one type is most effective.

### Limitations

While the observational design allowed us to analyze individuals’ language from real-time use of the program, the correlational design poses limitations. First, it is unknown whether language is independently exerting effect, as in the experimental studies described above, or whether it exerts an indirect effect by solely acting as a proxy for other psychological factors. Regardless of the causal role of language, this particular study sought to explore if spontaneously written goal language gathered from actual use of the program is associated with attrition and weight loss. Future studies should extend these findings by investigating mediating and moderating relationships between language variables and other demographic and psychological characteristics. For example, does educational attainment or socioeconomic status moderate the relationships shown here? There is initial evidence suggesting that distanced language is positively correlated with educational achievement and socioeconomic status [21, 72]. This is thought to be because distanced writing involves writing, and thus thinking, in an analytical and abstract manner [21]. In the present study, we controlled for demographic differences between participants in attrition (age) and for both age and baseline BMI in weight analyses, but future work should control for demographics such as educational attainment or socioeconomic status, which could conceivably be related to one’s tendency to use either distanced and analytical or immediate and narrative language. A psychological factor that may be important to consider is construal level. As previously described, a low level goal construal focuses on concrete details (e.g., this food smells good), while a high level construal of goals focuses on broader motivations to perform a behavior (this food is healthy) [27]. Earlier, we speculated that this is why distanced language is associated with less attrition and better weight loss. Future research should examine whether construal level mediates, and ultimately explains, the relationships between distanced or immediate language and outcomes. We also used a self-selected sample of individuals who voluntarily joined this program. This is not representative of all individuals who might intentionally lose weight. Future studies could consider adding writing prompts to general population surveys or studies. For example, if general population surveys of intentional weight management are conducted longitudinally, open-ended fields could be added in which individuals are asked to write down all of their relevant thoughts for 15 minutes (see [15] for example prompts). Another limitation is that self-reported weight was used, rather than weight measurements that were recorded and validated in person. Future studies should examine if the findings replicate with objective measurement of weight. Finally, it should be noted that the LIWC program uses pre-defined categories that, like all closed dictionary approaches, may not accurately classify complex contexts such as sarcasm. Future research should use analysis of closed and open approaches to investigate language in attrition and weight loss. Open approaches, while computationally intensive, leverage machine-learning and natural language processing to enable learning patterns based on words often used together [72].

### Conclusion

To our knowledge, this is the first study to show that the language used on a weight management program is associated with outcomes. Thus, our results are among the first to identify individuals’ language, which has not been studied much previously, as relevant and informative for weight management interventions. This raises directions for future research to improve intervention development and ascertain whether language is informative in other lifestyle behavior change interventions. Our results suggest that psychologically distanced language, and in particular, immediate language, are most informative. In both univariate and multivariate models, immediate language markers such as personal pronouns, discrepancy words, and auxiliary verbs were associated with attrition, and personal pronouns, discrepancy words, and future tense words were associated with weight loss. In terms of goal setting, tentative words for attrition or positive emotion and insight words for weight loss could be informative, but future work should corroborate these results. Future work should examine whether individuals assigned to write in these ways could experience improved outcomes, the feasibility and effectiveness of just-in-time triggers to write with more distance, and the effects of distanced writing compared to writing naturally or immediate writing. A next step for future research is also to examine how language compares to other common psychological factors, such as conscientiousness, that can only be measured via self-report, as well as to explore how to combine language and self-reported measurements most effectively.

## Supporting information

S1 Text Table A. Goal setting: All univariate age-adjusted associations between language categories and attrition (logistic regressions). Table B. Goal setting: All univariate age and baseline BMI-adjusted associations between language categories and weight loss (linear regressions). Table C. Goal striving: All univariate age-adjusted associations between language categories and attrition (logistic regressions). Table D. Goal striving: multivariate age-adjusted associations between language categories and attrition/weight loss during goal striving (logistic regression and linear regression). Table E. Goal striving: All age and baseline BMI-adjusted associations between language categories and weight loss (linear regressions).
(DOC)

## References

[1] Hales CM, Fryar CD, Carroll MD, Freedman DS, Ogden CL. Trends in Obesity and Severe Obesity Prevalence in US Youth and Adults by Sex and Age, 2007–2008 to 2015–2016. JAMA. 2018 Apr 24;319(16):1723–5. doi: 10.1001/jama.2018.3060 29570750 PMC5876828

[2] Pi-Sunyer FX. The obesity epidemic: pathophysiology and consequences of obesity. Obes Res. 2002 Dec;10 Suppl 2:97S–104S. doi: 10.1038/oby.2002.202 12490658

[3] Goldstein DJ. Beneficial health effects of modest weight loss. International journal of obesity and related metabolic disorders: journal of the International Association for the Study of Obesity. 1992;16(6):397–415. 1322866

[4] Ryan DH, Yockey SR. Weight Loss and Improvement in Comorbidity: Differences at 5%, 10%, 15%, and Over. Curr Obes Rep. 2017 Jun;6(2):187–94. doi: 10.1007/s13679-017-0262-y 28455679 PMC5497590

[5] Wing RR. Behavioral weight control. In: Wadden TA, Stunkard AJ, editors. Handbook of Obesity Treatment. Guilford Press; 2002. p. 301–16.

[6] Moroshko I, Brennan L, O’Brien P. Predictors of dropout in weight loss interventions: a systematic review of the literature: Dropout in weight loss interventions. Obesity Reviews. 2011 Nov;12(11):912–34. doi: 10.1111/j.1467-789X.2011.00915.x 21815990

[7] Miller BML, Brennan L. Measuring and reporting attrition from obesity treatment programs: A call to action! Obesity Research & Clinical Practice. 2015 May;9(3):187–202. doi: 10.1016/j.orcp.2014.08.007 25293585

[8] Chopra S, Malhotra A, Ranjan P, Vikram NK, Sarkar S, Siddhu A, et al. Predictors of successful weight loss outcomes amongst individuals with obesity undergoing lifestyle interventions: A systematic review. Obesity Reviews [Internet]. 2021 Mar [cited 2021 Apr 12];22(3). Available from: https://onlinelibrary.wiley.com/doi/10.1111/obr.13148 33200547 10.1111/obr.13148

[9] Stubbs J, Whybrow S, Teixeira P, Blundell J, Lawton C, Westenhoefer J, et al. Problems in identifying predictors and correlates of weight loss and maintenance: implications for weight control therapies based on behaviour change: Predicting weight outcomes. Obesity Reviews. 2011 May;12(9):688–708. doi: 10.1111/j.1467-789X.2011.00883.x 21535362

[10] Nahum-Shani I, Smith SN, Spring BJ, Collins LM, Witkiewitz K, Tewari A, et al. Just-in-Time Adaptive Interventions (JITAIs) in Mobile Health: Key Components and Design Principles for Ongoing Health Behavior Support. Annals of Behavioral Medicine. 2018 May 18;52(6):446–62. doi: 10.1007/s12160-016-9830-8 27663578 PMC5364076

[11] Schembre SM, Liao Y, Robertson MC, Dunton GF, Kerr J, Haffey ME, et al. Just-in-Time Feedback in Diet and Physical Activity Interventions: Systematic Review and Practical Design Framework. Journal of Medical Internet Research. 2018 Mar 22;20(3):e8701. doi: 10.2196/jmir.8701 29567638 PMC5887039

[12] Forman EM, Goldstein SP, Crochiere RJ, Butryn ML, Juarascio AS, Zhang F, et al. Randomized controlled trial of OnTrack, a just-in-time adaptive intervention designed to enhance weight loss. Translational Behavioral Medicine. 2019 Nov 25;9(6):989–1001. doi: 10.1093/tbm/ibz137 31602471

[13] Crochiere RJ, Zhang F (Zoe), Juarascio AS, Goldstein SP, Thomas JG, Forman EM. Comparing ecological momentary assessment to sensor-based approaches in predicting dietary lapse. Translational Behavioral Medicine. 2021 Dec 1;11(12):2099–109. doi: 10.1093/tbm/ibab123 34529044

[14] Goldstein SP, Hoover A, Evans EW, Thomas JG. Combining ecological momentary assessment, wrist-based eating detection, and dietary assessment to characterize dietary lapse: A multi-method study protocol. Digital Health. 2021 Jan 1;7:2055207620988212. doi: 10.1177/2055207620988212 33598309 PMC7863144

[15] Pennebaker JW. Writing about Emotional Experiences as a Therapeutic Process. Psychological Science. 1997;8(3):162–6.

[16] Pennebaker JW. Telling stories: the health benefits of narrative. Lit Med. 2000;19(1):3–18. doi: 10.1353/lm.2000.0011 10824309

[17] Kornfield R, Toma CL, Shah DV, Moon TJ, Gustafson DH. What Do You Say Before You Relapse? How Language Use in a Peer-to-Peer Online Discussion Forum Predicts Risky Drinking Among Those in Recovery. Health Commun. 2018 Sep;33(9):1184–93. doi: 10.1080/10410236.2017.1350906 28792228 PMC6059378

[18] Ziemer KS, Korkmaz G. Using text to predict psychological and physical health: A comparison of human raters and computerized text analysis. Computers in Human Behavior. 2017 Nov 1;76:122–7.

[19] Chung CK, Jones C, Liu A, Pennebaker JW. Predicting Success and Failure in Weight Loss Blogs through Natural Language Use. Proceedings of the 2^nd^ annual International Conference on Weblogs and Social Media; 2008 Mar 30-Apr 2; Seattle, Washington, USA.

[20] Pennebaker JW. The secret life of pronouns: What our words say about us. London, England: Bloomsbury Press; 2011.

[21] Pennebaker JW, Chung CK, Frazee J, Lavergne GM, Beaver DI. When Small Words Foretell Academic Success: The Case of College Admissions Essays. PLOS ONE. 2014 Dec 31;9(12):e115844. doi: 10.1371/journal.pone.0115844 25551217 PMC4281205

[22] Tausczik YR, Pennebaker JW. The Psychological Meaning of Words: LIWC and Computerized Text Analysis Methods. Journal of Language and Social Psychology. 2010 Mar 1;29(1):24–54.

[23] Nook E, Schleider J, Somerville L. A Linguistic Signature of Psychological Distancing in Emotion Regulation. Journal of Experimental Psychology General. 2017 Mar 3;146:337–46. doi: 10.1037/xge0000263 28114772

[24] Shahane AD, Fagundes CP, Buka SL, Denny BT. Associations Between Linguistic Markers of Emotion Regulation and Cardiovascular Disease-Related Inflammation. Psychoneuroimmunology Journal. 2021;12(13):1–8.

[25] Shahane AD, Denny BT. Predicting emotional health indicators from linguistic evidence of psychological distancing. Stress and Health. 2019;35(2):200–10. doi: 10.1002/smi.2855 30623579

[26] Nook EC, Stavish CM, Sasse SF, Lambert HK, Mair P, McLaughlin KA, et al. Charting the development of emotion comprehension and abstraction from childhood to adulthood using observer-rated and linguistic measures. Emotion. 2020 Aug;20(5):773–92. doi: 10.1037/emo0000609 31192665 PMC6908774

[27] Fujita K, Carnevale JJ. Transcending Temptation Through Abstraction: The Role of Construal Level in Self-Control. Curr Dir Psychol Sci. 2012 Aug;21(4):248–52.

[28] Huisman S, Maes S, De Gucht VJ, Chatrou M, Haak HR. Low Goal Ownership Predicts Drop-out from a Weight Intervention Study in Overweight Patients with Type 2 Diabetes. Int J Behav Med. 2010;17(3):176–81. doi: 10.1007/s12529-009-9071-3 20033629 PMC2910303

[29] Burgess E, Hassmén P, Pumpa KL. Determinants of adherence to lifestyle intervention in adults with obesity: a systematic review. Clinical Obesity. 2017;7(3):123–35. doi: 10.1111/cob.12183 28296261

[30] Schippers MC, Morisano D, Locke EA, Scheepers AWA, Latham GP, de Jong EM. Writing about personal goals and plans regardless of goal type boosts academic performance. Contemporary Educational Psychology. 2020 Jan 1;60:101823.

[31] King LA. The Health Benefits of Writing about Life Goals. Pers Soc Psychol Bull. 2001 Jul;27(7):798–807.

[32] Schmeichel BJ, Vohs KD, Duke SC. Self-Control at High and Low Levels of Mental Construal. Social Psychological and Personality Science. 2011 Mar 1;2(2):182–9.

[33] Park J, Hedgcock WM. Thinking concretely or abstractly: The influence of fit between goal progress and goal construal on subsequent self-regulation. Journal of Consumer Psychology. 2016;26(3):395–409.

[34] Locke EA, Latham GP. Building a practically useful theory of goal setting and task motivation: A 35-year odyssey. American Psychologist. 2002 Sep;57(9):705–17. doi: 10.1037//0003-066x.57.9.705 12237980

[35] Mann T, De Ridder D, Fujita K. Self-regulation of health behavior: social psychological approaches to goal setting and goal striving. Health Psychology. 2013;32(5):487. doi: 10.1037/a0028533 23646832

[36] Behr H, Ho AS, Mitchell ES, Yang Q, DeLuca L, Michealides A. How Do Emotions during Goal Pursuit in Weight Change over Time? Retrospective Computational Text Analysis of Goal Setting and Striving Conversations with a Coach during a Mobile Weight Loss Program. International Journal of Environmental Research and Public Health. 2021;18(12):6600. doi: 10.3390/ijerph18126600 34205282 PMC8296374

[37] Chung CK. Predicting weight loss in blogs using computerized text analysis [dissertation]. Austin, TX: University of Texas Austin; 2009.

[38] Lindstrom Johnson S, Blum RW, Cheng TL. Future Orientation: A Construct with Implications for Adolescent Health and Wellbeing. Int J Adolesc Med Health. 2014;26(4):459–68. doi: 10.1515/ijamh-2013-0333 24523304 PMC4827712

[39] Cho SMJ, Lee JH, Shim J-S, Yeom H, Lee SJ, Jeon YW, et al. Effect of Smartphone-Based Lifestyle Coaching App on Community-Dwelling Population With Moderate Metabolic Abnormalities: Randomized Controlled Trial. Journal of Medical Internet Research. 2020 Oct 9;22(10):e17435. doi: 10.2196/17435 33034564 PMC7584978

[40] DeLuca L, Toro-Ramos T, Michaelides A, Seng E, Swencionis C. Relationship Between Age and Weight Loss in Noom: Quasi-Experimental Study. JMIR Diabetes. 2020 Jun 4;5(2):e18363. doi: 10.2196/18363 32497017 PMC7303833

[41] Toro-Ramos T, Michaelides A, Anton M, Karim Z, Kang-Oh L, Argyrou C, et al. Mobile Delivery of the Diabetes Prevention Program in People With Prediabetes: Randomized Controlled Trial. JMIR mHealth and uHealth. 2020;8(7):e17842. doi: 10.2196/17842 32459631 PMC7381044

[42] Lee K-W, Kim H-B, Lee S-H, Ha H-K. Changes in Weight and Health-Related Behavior Using Smartphone Applications in Patients With Colorectal Polyps. Journal of Nutrition Education and Behavior. 2019 May;51(5):539–46. doi: 10.1016/j.jneb.2019.02.002 30902428

[43] Castelnuovo G, Pietrabissa G, Manzoni GM, Cattivelli R, Rossi A, Novelli M, et al. Cognitive behavioral therapy to aid weight loss in obese patients: current perspectives. PRBM. 2017 Jun;Volume 10:165–73. doi: 10.2147/PRBM.S113278 28652832 PMC5476722

[44] Armstrong M, Mottershead T, Ronksley P, Sigal R, Campbell T, Hemmelgarn B. Motivational interviewing to improve weight loss in overweight and/or obese patients: a systematic review and meta-analysis of randomized controlled trials. Obesity reviews. 2011;12(9):709–23. doi: 10.1111/j.1467-789X.2011.00892.x 21692966

[45] Lawlor ER, Islam N, Bates S, Griffin SJ, Hill AJ, Hughes CA, et al. Third-wave cognitive behaviour therapies for weight management: A systematic review and network meta-analysis. Obesity Reviews. 2020;21(7):e13013. doi: 10.1111/obr.13013 32181957 PMC7379202

[46] Pennebaker JW, Chung CK. Counting little words in big data: The psychology of individuals, communities, culture, and history. In: Forgas JP, Vincze O, & László J, editors. Social cognition and communication. New York, NY, US: Psychology Press; 2014. p. 25–42.

[47] Benjamini Y, Hochberg Y. Controlling the false discovery rate: a practical and powerful approach to multiple testing. Journal of the Royal statistical society: series B (Methodological). 1995;57(1):289–300.

[48] Appelhans BM, French SA, Pagoto SL, Sherwood NE. Managing temptation in obesity treatment: a neurobehavioral model of intervention strategies. Appetite. 2016 Jan 1;96:268–79. doi: 10.1016/j.appet.2015.09.035 26431681 PMC4684710

[49] Swinburn B, Egger G, Raza F. Dissecting obesogenic environments: the development and application of a framework for identifying and prioritizing environmental interventions for obesity. Prev Med. 1999 Dec;29(6 Pt 1):563–70. doi: 10.1006/pmed.1999.0585 10600438

[50] Papies EK. Health goal priming as a situated intervention tool: how to benefit from nonconscious motivational routes to health behaviour. Health Psychol Rev. 2016 Oct 1;10(4):408–24. doi: 10.1080/17437199.2016.1183506 27144729 PMC5214881

[51] Miller WC, Koceja DM, Hamilton EJ. A meta-analysis of the past 25 years of weight loss research using diet, exercise or diet plus exercise intervention. Int J Obes Relat Metab Disord. 1997 Oct;21(10):941–7. doi: 10.1038/sj.ijo.0800499 9347414

[52] Catenacci VA, Wyatt HR. The role of physical activity in producing and maintaining weight loss. Nat Rev Endocrinol. 2007 Jul;3(7):518–29. doi: 10.1038/ncpendmet0554 17581621 PMC4578965

[53] Daniel TO, Stanton CM, Epstein LH. The future is now: Comparing the effect of episodic future thinking on impulsivity in lean and obese individuals. Appetite. 2013 Dec 1;71:120–5. doi: 10.1016/j.appet.2013.07.010 23917063 PMC4185182

[54] Ireland ME, Schwartz HA, Chen Q, Ungar L, Albarracín D. Future-Oriented Tweets Predict Lower County-Level HIV Prevalence in the United States. Health Psychol. 2015 Dec;34 Suppl:1252–60. doi: 10.1037/hea0000279 26651466 PMC5621637

[55] Barlow P, Reeves A, McKee M, Galea G, Stuckler D. Unhealthy diets, obesity and time discounting: a systematic literature review and network analysis. Obesity reviews. 2016 Sep;17(9):810–9. doi: 10.1111/obr.12431 27256685 PMC4988386

[56] Amlung M, Petker T, Jackson J, Balodis I, MacKillop J. Steep discounting of delayed monetary and food rewards in obesity: a meta-analysis. Psychological medicine. 2016 Aug;46(11):2423–34. doi: 10.1017/S0033291716000866 27299672

[57] Stein JS, Sze YY, Athamneh L, Koffarnus MN, Epstein LH, Bickel WK. Think fast: rapid assessment of the effects of episodic future thinking on delay discounting in overweight/obese participants. Journal of Behavioral Medicine. 2017 Oct;40(5):832–8. doi: 10.1007/s10865-017-9857-8 28508382 PMC5685941

[58] Demiray B, Mehl MR, Martin M. Conversational time travel: Evidence of a retrospective bias in real life conversations. Frontiers in Psychology. 2018:2160. doi: 10.3389/fpsyg.2018.02160 30483183 PMC6243041

[59] Jeffery RW, Linde JA, Finch EA, Rothman AJ, King CM. A Satisfaction Enhancement Intervention for Long-Term Weight Loss. Obesity. 2006;14(5):863–9. doi: 10.1038/oby.2006.100 16855196

[60] Foster GD, Wadden TA, Vogt RA, Brewer G. What is a reasonable weight loss? Patients’ expectations and evaluations of obesity treatment outcomes. Journal of Consulting and Clinical Psychology. 1997;65(1):79–85. doi: 10.1037//0022-006x.65.1.79 9103737

[61] Dombrowski SU, Endevelt R, Steinberg DM, Benyamini Y. Do more specific plans help you lose weight? Examining the relationship between plan specificity, weight loss goals, and plan content in the context of a weight management programme. Br J Health Psychol. 2016 Nov;21(4):989–1005. doi: 10.1111/bjhp.12212 27454908

[62] Alfonsson S, Olsson E, Hursti T. Motivation and Treatment Credibility Predicts Dropout, Treatment Adherence, and Clinical Outcomes in an Internet-Based Cognitive Behavioral Relaxation Program: A Randomized Controlled Trial. Journal of Medical Internet Research. 2016 Mar 8;18(3):e5352. 10.2196/jmir.5352 PMC4804106 26957354

[63] Richard M, Christina MF, Deborah LS, Rubio N, Kennon MS. Intrinsic motivation and exercise adherence. Int J Sport Psychol. 1997;28(4):335–54.

[64] Abe JAA. Words that predict outstanding performance. Journal of Research in Personality. 2009 Jun;43(3):528–31.

[65] Dennis JP, Vander Wal JS. The Cognitive Flexibility Inventory: Instrument Development and Estimates of Reliability and Validity. Cogn Ther Res. 2010 Jun;34(3):241–53.

[66] Zare H, Baradaran M. Effectiveness of Acceptance and Commitment Therapy (ACT) in improving of cognitive control and cognitive flexibility in anxious students. Quarterly of Applied Psychology. 2019;12(4):491–511.

[67] Raman J, Hay P, Tchanturia K, Smith E. A randomised controlled trial of manualized cognitive remediation therapy in adult obesity. Appetite. 2018 Apr 1;123:269–79. doi: 10.1016/j.appet.2017.12.023 29278718

[68] Teixeira PJ, Silva MN, Coutinho SR, Palmeira AL, Mata J, Vieira PN, et al. Mediators of Weight Loss and Weight Loss Maintenance in Middle-aged Women. Obesity. 2010;18(4):725–35. doi: 10.1038/oby.2009.281 19696752

[69] Kaye BK. It’s a Blog, Blog, Blog World: Users and Uses of Weblogs. Atlantic Journal of Communication. 2005 Jun 1;13(2):73–95.

[70] Dogra V, Verma M. Communication for affiliation: An indicative approach to understand blogging. Amity Journal of Media and Communication Studies. 2011;63.

[71] Hine M, Nardon L, Gulanowski D. The Role of Emotional Expression in Accessing Social Networks: The Case of Newcomers’ Blogs. Journal of International Technology and Information Management. 2019 May 1;28(1):29–51.

[72] Alvero AJ, Giebel S, Gebre-Medhin B, Antonio AL, Stevens ML, Domingue BW. Essay content and style are strongly related to household income and SAT scores: Evidence from 60,000 undergraduate applications. Science advances. 2021 Oct 13;7(42):eabi9031. doi: 10.1126/sciadv.abi9031 34644119 PMC8514086

