# Supplementary material for: Goal language is associated with attrition and weight loss on a digital program: Observational study
Source: PLOS Digit Health. 2022 Jun 16;1(6):e0000050. doi: 10.1371/journal.pdig.0000050 (PMC9931249; doi:10.1371/journal.pdig.0000050)
Supplement: S1 Text — Table A. Goal setting: All univariate age-adjusted associations between language categories and attrition (logistic regressions). Table B. Goal setting: All univariate age and baseline BMI-adjusted associations between language categories and weight loss (linear regressions). Table C. Goal striving: All univariate age-adjusted associations between language categories and attrition (logistic regressions). Table D. Goal striving: multivariate age-adjusted associations between language categories and attrition/weight loss during goal striving (logistic regression and linear regression). Table E. Goal striving: All age and baseline BMI-adjusted associations between language categories and weight loss (linear regressions). (DOC) [file pdig.0000050.s001.doc]

**Appendix**

**Table A.** Goal setting: All univariate age-adjusted associations between language categories and attrition (logistic regressions).

| **Covariate** | **Related concept** | **B** | **SE** | **Wald** | ***p*** | **OR** | **95% CI** |
| --- | --- | --- | --- | --- | --- | --- | --- |
| Articles | Distanced (vs. immediate) language | -0.03 | 0.02 | -1.64 | 0.10 | 0.97 | (0.94, 1.00) |
| Prepositions | Distanced (vs. immediate) language | -0.007 | 0.008 | -0.84 | 0.40 | 0.99 | (0.98, 1.01) |
| Auxiliary verbs | Immediate (vs. distanced) language | -0.001 | 0.01 | -0.08 | 0.94 | 1.00 | (0.98, 1.02) |
| Personal pronouns | Immediate (vs. distanced) language | -0.015 | 0.009 | -1.73 | 0.08 | 0.98 | (0.97,1.00) |
| Present tense | Immediate (vs. distanced) language | -0.001 | 0.007 | -0.14 | 0.89 | 1.00 | (0.98, 1.01) |
| Discrepancy words | Immediate (vs. distanced) language | -0.03 | 0.02 | -1.41 | 0.16 | 0.97 | (0.94, 1.01) |
| Positive emotion | Emotion | 0.004 | 0.008 | 0.55 | 0.58 | 1.004 | (0.99, 1.02) |
| Negative emotion | Emotion | 0.006 | 0.012 | 0.46 | 0.65 | 1.006 | (0.98, 1.09) |
| Insight words | Cognitive processes | 0.02 | 0.01 | 1.34 | 0.18 | 1.02 | (0.99, 1.04) |
| Causal words | Cognitive processes | -0.001 | 0.02 | -0.06 | 0.95 | 1.00 | (0.97, 1.03) |
| Tentative words | Cognitive processes | -0.09 | 0.03 | -2.78 | 0.005** | 0.91 | (0.86, 0.97) |
| Certainty words | Cognitive processes | 0.02 | 0.02 | 1.08 | 0.28 | 1.02 | (0.98, 1.05) |
| Differentiating words | Cognitive processes | -0.02 | 0.02 | -1.01 | 0.31 | 0.97 | (0.93, 1.02) |
| Ingestion words | Food/Weight | 0.002 | 0.02 | 0.13 | 0.90 | 1.002 | (0.97, 1.04) |
| Social words | Social relationships | -0.006 | 0.01 | -0.51 | 0.61 | 0.99 | (0.97, 1.02) |
| Future tense | Future goal orientation | 0.003 | 0.03 | 0.09 | 0.92 | 1.00 | (0.95, 1.06) |

Note: Attrition was coded as 0 (retained) 1 (attrited). * denotes significance without multiple comparison adjustment, ** denotes significance with multiple comparison adjustment

**Table B.** Goal setting: All univariate age and baseline BMI-adjusted associations between language categories and weight loss (linear regressions).

| **Covariate** | **Related concept** | **B (95% CI)** | **SE** | ***t*** | ***p*** | **Cohen’s f2** |
| --- | --- | --- | --- | --- | --- | --- |
| Articles | Distanced (vs. immediate) language | -0.01 (-0.09, 0.06) | 0.04 | -0.41 | .68 | 0.0001 |
| Prepositions | Distanced (vs. immediate) language | 0.04 (0.002, 0.07) | 0.02 | 2.06 | .04* | 0.004 |
| Auxiliary verbs | Immediate (vs. distanced) language | 0.04 (-0.003, 0.08) | 0.02 | 1.80 | .07 | 0.003 |
| Personal pronouns | Immediate (vs. distanced) language | 0.02 (-0.02, 0.05) | 0.02 | 0.95 | .34 | 0.0009 |
| Present tense | Immediate (vs. distanced) language | -0.001 (-0.03, 0.03) | 0.02 | -0.09 | .93 | <0.0001 |
| Discrepancy words | Immediate (vs. distanced) language | 0.002 (-0.08, 0.08) | 0.04 | 0.06 | .95 | <0.0001 |
| Positive emotion | Emotion | -0.04 (-0.08, -0.01) | 0.02 | -2.55 | .01* | 0.005 |
| Negative emotion | Emotion | 0.02 (-0.03, 0.07) | 0.03 | 0.64 | .52 | 0.0004 |
| Insight words | Cognitive processes | -0.07 (-0.12, -0.02) | 0.03 | -2.59 | .01* | 0.005 |
| Causal words | Cognitive processes | -0.03 (-0.10, 0.04) | 0.04 | -0.76 | .44 | 0.0004 |
| Tentative words | Cognitive processes | 0.11 (-0.02, 0.24) | 0.07 | 1.65 | .10 | 0.002 |
| Certainty words | Cognitive processes | -0.04 (-0.11, 0.03) | 0.04 | -1.08 | .28 | 0.0009 |
| Differentiating words | Cognitive processes | 0.10 (-0.007, 0.20) | 0.05 | 1.83 | .07 | 0.002 |
| Ingestion words | Food/Weight | -0.04 (-0.11, 0.03) | 0.04 | -1.18 | .24 | 0.0009 |
| Social words | Social relationships | 0.006 (-0.04, 0.05) | 0.02 | 0.24 | .81 | 0.0001 |
| Future tense | Future goal orientation | -0.04 (-0.16, 0.09) | 0.06 | -0.58 | .56 | 0.0004 |

Note: * denotes significance without multiple comparison adjustment, ** denotes significance with multiple comparison adjustment. A Cohen’s f2 of .02 is estimated to be a small effect size.

**Table C.** Goal striving: All univariate age-adjusted associations between language categories and attrition during goal striving (logistic regressions).

| **Covariate** | **Related concept** | **B** | **SE** | **Wald** | ***p*** | **OR** | **95% CI** |
| --- | --- | --- | --- | --- | --- | --- | --- |
| Articles | Distanced (vs. immediate) language | -0.15 | 0.04 | -3.72 | <.001** | 0.86 | (0.80, 0.93) |
| Prepositions | Distanced (vs. immediate) language | -0.04 | 0.02 | -1.53 | .13 | 0.96 | (0.91, 1.01) |
| Auxiliary verbs | Immediate (vs. distanced) language | 0.14 | 0.03 | 5.26 | <.001** | 1.15 | (1.09, 1.21) |
| Personal pronouns | Immediate (vs. distanced) language | 0.12 | 0.02 | 5.26 | <.001** | 1.13 | (1.08, 1.18) |
| Present tense | Immediate (vs. distanced) language | 0.09 | 0.02 | 4.15 | <.001** | 1.10 | (1.05, 1.15) |
| Discrepancy words | Immediate (vs. distanced) language | 0.24 | 0.06 | 4.00 | <.001** | 1.27 | (1.13, 1.43) |
| Positive emotion | Emotion | -0.003 | 0.03 | -0.14 | .89 | 1.00 | (0.95, 1.05) |
| Negative emotion | Emotion | -0.004 | 0.06 | -0.07 | .95 | 1.00 | (0.88, 1.12) |
| Insight words | Cognitive processes | -0.05 | 0.05 | -1.05 | .30 | 0.95 | (0.87, 1.04) |
| Causal words | Cognitive processes | -0.03 | 0.06 | -0.53 | .59 | 0.97 | (0.87, 1.08) |
| Tentative words | Cognitive processes | -0.11 | 0.05 | -2.49 | .01* | 0.89 | (0.81, 0.97) |
| Certainty words | Cognitive processes | 0.01 | 0.07 | 0.19 | .85 | 1.01 | (0.88, 1.17) |
| Differentiating words | Cognitive processes | 0.04 | 0.04 | 0.98 | .33 | 1.04 | (0.96, 1.13) |
| Ingestion words | Food/Weight | -0.02 | 0.03 | -0.66 | .51 | 0.98 | (0.93, 1.03) |
| Social words | Social relationships | 0.04 | 0.03 | 1.26 | .21 | 1.04 | (0.98, 1.10) |
| Future tense | Future goal orientation | -0.14 | 0.06 | -2.26 | .02* | 0.87 | (0.77, 0.98) |

Note: * denotes significance without multiple comparison adjustment, ** denotes significance with multiple comparison adjustment

**Table D.** Goal striving: multivariate age-adjusted associations between language categories and attrition/weight loss during goal striving (logistic regression and linear regression).

| **Multivariate model for attrition** | | | | | | | |
| --- | --- | --- | --- | --- | --- | --- | --- |
| **Covariate** | **Related concept** | **B** | **SE** | **Wald** | ***p*** | **OR** | **95% CI** |
| Articles | Distanced (vs. immediate) language | -0.05 | 0.04 | -1.17 | .24 | 0.95 | (0.87, 1.04) |
| Auxiliary verbs | Immediate (vs. distanced) language | 0.10 | 0.03 | 3.43 | <.001 | 1.10 | (1.04, 1.17) |
| Personal pronouns | Immediate (vs. distanced) language | 0.06 | 0.03 | 2.18 | .03 | 1.06 | (1.01, 1.12) |
| Present tense | Immediate (vs. distanced) language | 0.14 | 0.03 | 0.51 | .61 | 1.01 | (0.96, 1.07) |
| Discrepancy words | Immediate (vs. distanced) language | 0.19 | 0.06 | 3.01 | .003 | 1.21 | (1.07, 1.38) |
| Age | Covariate | -0.02 | 0.005 | -3.69 | <.001 | 0.98 | (0.97, 0.99) |
| **Multivariate model for weight loss** | | | | | | | |
| **Covariate** | **Related concept** | **B (95% CI)** | **SE** | ***t*** | ***p*** |  |  |
| Articles | Distanced (vs. immediate) language | 0.06 | 0.09 | 0.70 | .48 |  |  |
| Prepositions | Distanced (vs. immediate) language | 0.07 | 0.05 | 1.35 | .18 |  |  |
| Auxiliary verbs | Immediate (vs. distanced) language | -0.03 | 0.05 | -0.61 | .54 |  |  |
| Personal pronouns | Immediate (vs. distanced) language | -0.11 | 0.05 | -2.14 | .03 |  |  |
| Present tense | Immediate (vs. distanced) language | -0.06 | 0.05 | -1.29 | .20 |  |  |
| Discrepancy words | Immediate (vs. distanced) language | -0.24 | 0.12 | -2.10 | .04 |  |  |
| Future tense | Future goal orientation | 0.32 | 0.12 | 2.56 | .01 |  |  |
| Age | Covariate | 0.04 | 0.001 | 3.78 | <.001 |  |  |
| Initial BMI | Covariate | 0.09 | 0.01 | 6.42 | <.001 |  |  |

**Table E.** Goal striving: All age and baseline BMI-adjusted associations between language categories and weight loss during goal striving conversations (linear regressions).

| **Covariate** | **Related concept** | **B (95% CI)** | **SE** | ***t*** | ***p*** | **Cohen’s f2** |
| --- | --- | --- | --- | --- | --- | --- |
| Articles | Distanced (vs. immediate) language | 0.26 (0.10, 0.41) | 0.08 | 3.29 | .001** | 0.01 |
| Prepositions | Distanced (vs. immediate) language | 0.17 (0.07, 0.27) | 0.05 | 3.33 | <.001** | 0.01 |
| Auxiliary verbs | Immediate (vs. distanced) language | -0.15 (-0.24, -0.06) | 0.05 | -3.21 | .001** | 0.01 |
| Personal pronouns | Immediate (vs. distanced) language | -0.23 (-0.31, -0.14) | 0.04 | -5.15 | <.001** | 0.03 |
| Present tense | Immediate (vs. distanced) language | -0.17 (-0.26, -0.09) | 0.04 | -4.04 | <.001** | 0.01 |
| Discrepancy words | Immediate (vs. distanced) language | -0.40 (-0.62, -0.17) | 0.11 | -3.47 | <.001** | 0.01 |
| Positive emotion | Emotion | 0.04 (-0.06, 0.15) | 0.05 | 0.84 | .40 | 0.0009 |
| Negative emotion | Emotion | -0.05 (-0.30, 0.19) | 0.12 | -0.44 | .66 | 0.0001 |
| Insight words | Cognitive processes | -0.008 (-.20, 0.18) | 0.10 | -0.08 | .93 | <0.00001 |
| Causal words | Cognitive processes | -0.001 (-0.22, 0.22) | 0.001 | 0.001 | .99 | <0.00001 |
| Tentative words | Cognitive processes | 0.14 (-0.05, 0.32) | 0.09 | 1.47 | .14 | 0.002 |
| Certainty words | Cognitive processes | 0.12 (-0.17, 0.41) | 0.15 | 0.82 | .41 | 0.0004 |
| Differentiating words | Cognitive processes | -0.09 (-0.26, 0.08) | 0.09 | -1.06 | .29 | 0.0009 |
| Ingestion words | Food/Weight | -0.05 (-0.15, 0.05) | 0.05 | -0.94 | .34 | 0.0009 |
| Social words | Social relationships | -0.11 (-0.23, 0.006) | 0.06 | -1.86 | .06 | 0.004 |
| Future tense | Future goal orientation | 0.45 (0.20, 0.69) | 0.12 | 3.61 | <.001** | 0.01 |

Note: * denotes significance without multiple comparison adjustment, ** denotes significance with multiple comparison adjustment. A Cohen’s f2 of .02 is estimated to be a small effect size.
